# Conceptual Perspectives: Bacterial Antimicrobial Peptide Induction as a Novel Strategy for Symbiosis with the Human Host

**DOI:** 10.3389/fmicb.2018.00302

**Published:** 2018-02-26

**Authors:** Santosh K. Ghosh, Zhimin Feng, Hisashi Fujioka, Renate Lux, Thomas S. McCormick, Aaron Weinberg

**Affiliations:** ^1^Biological Sciences, School of Dental Medicine, Case Western Reserve University, Cleveland, OH, United States; ^2^Electron Microscopy Core, School of Medicine, Case Western Reserve University, Cleveland, OH, United States; ^3^School of Dentistry, University of California, Los Angeles, Los Angeles, CA, United States; ^4^Department of Dermatology, School of Medicine, Case Western Reserve University, Cleveland, OH, United States

**Keywords:** *F. nucleatum*, *P. gingivalis*, symbiosis, beta-defensin, FAD-I

## Abstract

Human beta defensins (hBDs) are small cationic peptides, expressed in mucosal epithelia and important agents of innate immunity, act as antimicrobial and chemotactic agents at mucosal barriers. In this perspective, we present evidence supporting a novel strategy by which the oral bacterium *Fusobacterium nucleatum* induces hBDs and other antimicrobial peptides (AMPs) in normal human oral epithelial cells (HOECs) and thereby protects them from other microbial pathogens. The findings stress (1) the physiological importance of hBDs, (2) that this strategy may be a mechanism that contributes to homeostasis and health in body sites constantly challenged with bacteria and (3) that novel properties identified in commensal bacteria could, one day, be harnessed as new probiotic strategies to combat colonization of opportunistic pathogens. With that in mind, we highlight and review the discovery and characterization of a novel lipo-protein, FAD-I (*F**usobacterium* Associated Defensin Inducer) associated with the outer membrane of *F. nucleatum* that may act as a homeostatic agent by activating endogenous AMPs to re-equilibrate a dysregulated microenvironment. FAD-I has the potential to reduce dysbiosis-driven diseases at a time when resistance to antibiotics is increasing. We therefore postulate that FAD-I may offer a new paradigm in immunoregulatory therapeutics to bolster host innate defense of vulnerable mucosae, while maintaining physiologically responsive states of inflammation.

## Introduction

Symbiosis is a relationship between two organisms; it can be mutualistic where both the bacteria and the host benefit, or commensalistic where the bacteria benefits while the host is unharmed. The vast majority of the bacteria that resides in the human oral cavity, nose, throat, intestines, and on the skin, are commensals. Most of them are associated with mucosal surfaces that exposed to the external environment. A staggering 10^14^ bacterial cells have developed habitats to thrive on our mucosal surfaces (Henderson and Wilson, 1998), however, very little is known about their interactions with the host that contribute to favorable outcomes. In some instances, there are discernible bacteria that contribute to health, while others that contribute to illness. In other instances, this is not clearly defined, with some bacteria playing both a good and bad role. Cases of mutualistic interactions have already been shown in the intestine, where immune tolerance and inactivation of pro-inflammatory responses is maintained by non-virulent *Salmonella* (Neish et al., 2000). These organisms suppress the production of inflammatory cytokine by preventing ubiquitination and degradation of I*k*B, thereby blocking the activation of NF*k*B dependent immune response genes (Neish et al., 2000). This is not meant to infer that suppression of normal inflammation is always a good thing, as dysbiosis promoting keystone pathogens in inflammatory bowel disease and periodontal disease promote immune subversion that may lead to exacerbation of disease (Hajishengallis, 2014, 2015; Hajishengallis and Lamont, 2016).

The conceptual perspective that we discuss herein focuses on a novel dynamic between specific commensal oral bacteria and the host. Specifically, we will explain how these organisms may have evolved to promote symbiosis by regulating expression of key innate immune agents emanating from the mucosae. These discoveries provide a potential new paradigm for understanding the role of specific microbes, how they may contribute to homeostasis at the mucosal interface and how exploiting homeostasis-promoting beneficial agents produced by these microbes may one day be used to bolster defenses and quell dysbiosis at vulnerable mucosal body sites.

## Introducing the players

Since the human oral microflora is quite complex, to understand how its' many constituents contribute to oral health or disease is still quite daunting. While there are multiple examples of various gram-positive bacterial species residing congruously with each other within the oral cavity (Moore et al., 1984; Socransky and Haffajee, 1992; Socransky et al., 1998; Roberts and Darveau, 2002; Lu et al., 2005), equally plentiful examples of oral bacteria antagonizing to gain an advantage microniche abound (Kolenbrander et al., 1985; Kolenbrander, 2000). We realize that while time honored classifications of certain oral pathogens as commensals may be an anathema to some, for the sake of our conceptual perspective, herein we classify *Fusobacterium nucleatum*, a gram-negative oral fusiform bacterium, associated with dental plaque formation (Kolenbrander and London, 1993) which is ubiquitous in both healthy and diseased oral sites (Lee et al., 1999), as the “good” commensal. In contrast, *Porphyromonas gingivalis*, the “bad” commensal is a gram-negative bacterial opportunist that, along with representatives of the genus *Prevotella*, comprises the second most common cause of human infection by anaerobic gram-negative *bacilli* (Sherris, 1994). *P. gingivalis* stands out as a major etiologic agent in the initiation of periodontal destruction (Socransky and Haffajee, 1992). It can subvert innate immune responses (Madianos et al., 1997), efficiently invades normal human oral epithelial cells (HOECs) (Lamont et al., 1995), and periodontal tissues (Rudney et al., 2001), and has been described as a keystone pathogen in promoting dysbiosis in the context of periodontal disease (Hajishengallis et al., 2012).

## The host response

Mucosal epithelum is seen as the first line of defense between the host and the environment, and disturbance of these barriers can lead to microbial invasion and subsequent inflammation. Interestingly, the oral cavity is exceedingly forgiving and resilient as continuous abrasions, cuts, bites, burns and surgical procedures that compromise the oral epithelial barrier rarely lead to serious local infections or bacteremia (Zasloff, 2002). Numerous molecules play pivotal roles in protecting the oral cavity from persistent microbial challenges emanating from epithelial barrier disruption; included among these molecules are a class of antimicrobial peptides (AMPs) referred to as defensins. In oral tissue, constitutively expressed human beta-defensin-1 (hBD-1) is localized in suprabasal stratified epithelium. HBD-2, which is usually regulated by the transcription factor NFkB, co-localizes with hBD-1 and is therefore, detected in similarly differentiated upper epithelial layers, consistent with the development of the stratified epithelial barrier (Lu et al., 2005; Kawsar et al., 2009). HBD-3, is not expressed in the upper differentiated regions of the oral mucosa under normal conditions (Kawsar et al., 2009). Instead, it compartmentalizes to the less-differentiated and more proliferative stratum basale (Lu et al., 2005; Kawsar et al., 2009).

## The perceptions

With caveats in place for interpreting results emanating from “one bug–one host cell” interactions that are conducted in controlled environments that often oversimplify the complex dynamics of the oral cavity, a number of key findings have been made in regards to oral bacterial “cross-talk” with host cells. New insights are emerging detailing what *Porphyromonas gingivalis*, the single most compelling periodontopathogenic bacterium known to date, is doing when encountering HOECs and the physiological dysbiosis this promotes (Hajishengallis et al., 2012). A notable difference, observed *in vivo*, between human oral and most of the other epithelia of the body is the expression of hBD-2. This beta-defensin is induced in response to infection or inflammation in most mucosal tissues (O'Neil et al., 1999). However, it is expressed “constitutively” in normal oral tissue; i.e., in the absence of localized inflammation (Dale et al., 2001; Jurevic et al., 2003; Pazgier et al., 2006; Yang et al., 2007). We hypothesize that, specific oral commensal bacteria, e.g., *F. nucleatum*, in-part, contribute to hBD-2 expression in healthy uninflamed oral mucosa through contact with the epithelium (Krisanaprakornkit et al., 2002; Gupta et al., 2010). In contrast, other opportunistic bacteria such as the periodontopathogen *P. gingivalis*, display stealth-like qualities when in contact with host epithelia (Darveau et al., 1998; Lamont and Jenkinson, 1998), including the lack of induction of β-defensins, as reported previously by us and others (Krisanaprakornkit et al., 2002; Carlisle et al., 2009; Gupta et al., 2010). We have shown that while *P. gingivalis* challenge of HOECs results in little hBD-2 mRNA induction, *F. nucleatum* induces significant hBD-2 expression (Krisanaprakornkit et al., 1998, 2000, 2002; Gupta et al., 2010). Low level inductions of hBD-2 by HOECs following interaction with *P. gingivalis* has been explained by the organism's unique LPS structures, which may possibly impairs epithelial recognition of *P. gingivalis* and inhibit subsequent expression of hBD-2 (Lu et al., 2009). Moreover, *P. gingivalis* produces proteases capable of degrading β-defensins (Carlisle et al., 2009) and could abrogate defensin-related innate immune functions.

We conclude that defensins are important in epithelial mucosal function and homeostasis. Based on selective oral commensal bacterial induction of hBD-2 and other key AMPs, we provide a novel perspective on how such bacteria may be promoting site specific health without concomitant pro-inflammation. For the sake of simplicity, our model states that: (1) a beneficial bacterium is one that promotes AMP (e.g., hBD-2) induction in epithelial cells, and is resistant to the AMP it induces; (2) a beneficial bacterium, by inducing AMPs, enables the host to protect itself from potential attack by pathogenic bacteria and; (3) a non-beneficial bacterium is one that inhibits the beneficial bacterium from inducing AMPs. This concept may become useful, not just in recognizing symbiotic organisms in niches of the human body, but also in identifying AMP-inducing agents produced from such organisms, that could be harnessed to benefit the host when needed.

## Proof of concept

### The beneficial bacterium is resistant to hBDs while the non-beneficial/pathogenic bacterium is susceptible

**Figure 1:**
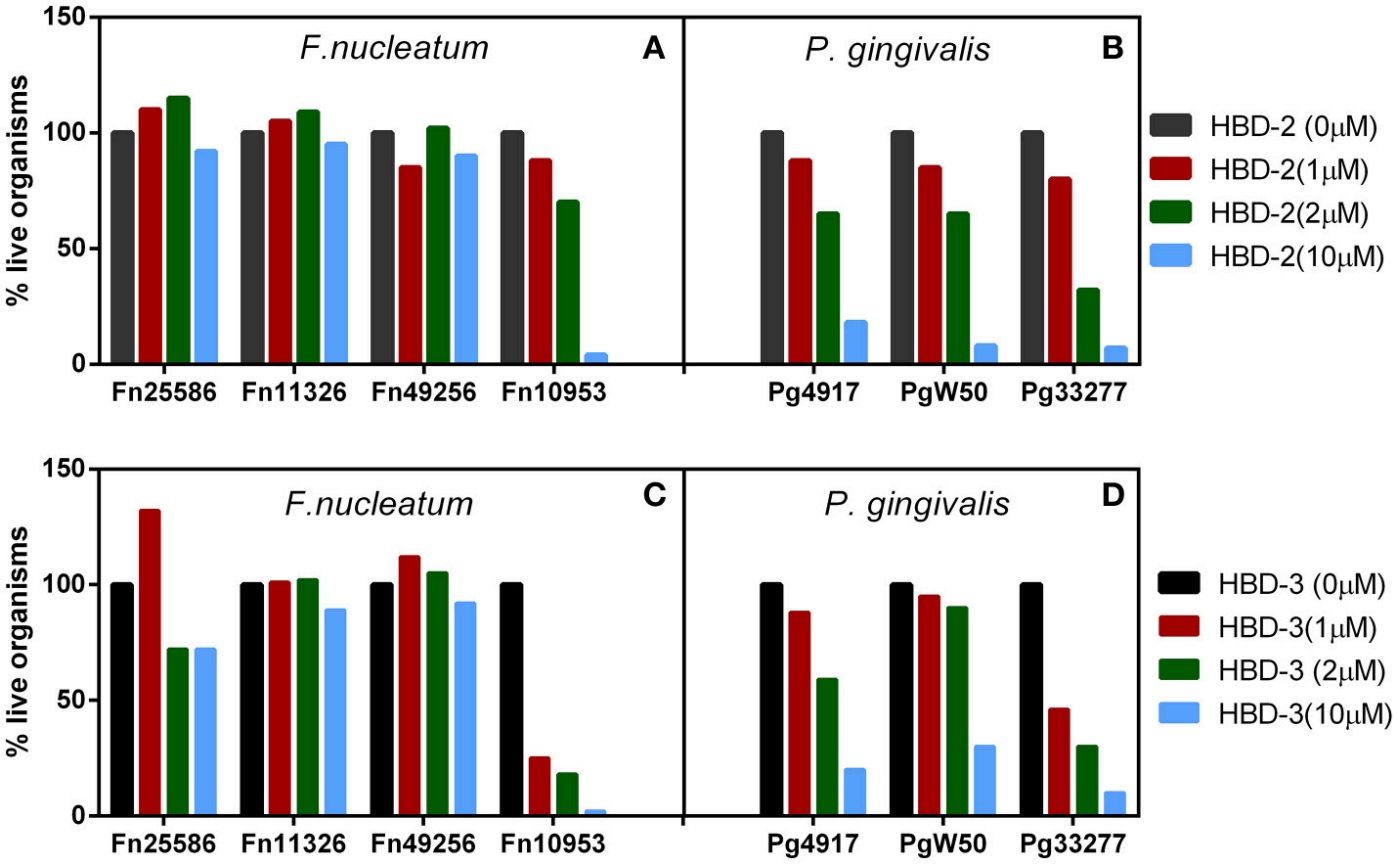
**(A–D)** Representative *F. nucleatum* **(A,C)** and *P. gingivalis* **(B,D)** susceptibility to hBD-2 **(A,B)** and−3 **(C,D)**, 2 × 10^5^ bacteria were incubated with recombinant hBD-2 and 3 (indicated micro-molar concentrations) anaerobically, for 3 h, followed by serial dilutions and plating on sheep red blood agar plates.

Recombinant forms of hBD-2 and −3 (rhBD) were generated (Harder et al., 2001; Ghosh et al., 2007) and tested for their ability to kill *F. nucleatum* and *P. gingivalis. In vitro* antimicrobial analysis revealed that while all three representative strains of *P. gingivalis* were killed by the hBDs (Figures 1B,D), at low micromolar concentrations and in a dose dependent manner, three out of the four *F. nucleatum* strains showed resistance (Figures 1A,C); i.e., strains 25586 and 23726 (subsp. *nucleatum*), as well as strain 49256 (subsp. *vincentii*). Interestingly, strain 10953 (subsp. *polymorphum*), which is a poor inducer of hBD-2 in HOECs (Bhattacharyya et al., 2016), is demonstrably sensitive to hBD-2 and hBD-3. Transmission electron microscopy of *F. nucleatum* type strain 25586 (subsp. *nucleatum*), which induces hBD-2 in HOECs (Krisanaprakornkit et al., 2002; Gupta et al., 2010), and is resistant to it (Figures 1A,C), revealed an extracellular factor that sequesters the cationic AMP away from the vulnerable anionic outer membrane of the bacterium. Interestingly, this factor was not found on the surface of *F. nucleatum* 10953 (data not shown), which could explain why this strain is susceptible to hBD-2 and−3. Could the extracellular factor be important, not only in resistance to hBDs, but also in inducing them? Could it be a novel mechanism whereby symbiotic *F. nucleatum* ssp. exert resistance by modifying their outer membranes, while other non-symbiotic *F. nucleatum* ssp. have not evolved to do so? Could this demonstrable difference be a reflection of co-evolution between certain members of *F. nucleatum* and the human host leading to symbiosis?

### The non-beneficial/pathogenic bacterium can inhibit the beneficial bacterium from inducing hBD's and other innate response elements

The non-beneficial bacterium, *P. gingivalis* demonstrates differential activation of inflammatory cytokines, when compared to the beneficial organism, *F. nucleatum. F. nucleatum* activates expression of IL-8, a potent PMN-inducing chemokine expressed by HOECs, and a possible reason for the presence of a constitutive IL-8 gradient in normal oral mucosa that contributes to entry of percolating PMNs into the oral mucosa (Darveau et al., 1998; Han et al., 2004; Quah et al., 2014). *P. gingivalis* not only inhibits HOEC IL-8 production directly, it shuts down the activation of IL-8 by *F. nucleatum* and other commensals (Madianos et al., 1997; Darveau et al., 1998; Li et al., 2015). Interestingly, Li et al. (2015) demonstrated that when HOECs were coinfected with both *F. nucleatum* and *P. gingivalis*, the latter repressed the activation of hBD-2 by *F. nucleatum*. Whether the inhibition of host innate immune response (e.g., induction of hBD-2) of *F. nucleatum* by *P. gingivalis*, is due to alteration of the host cell by *P. gingivalis* directly and/or to alteration in *F. nucleatum* that prevents hBD induction remains to be determined.

### The beneficial bacterium protects the host from the non-beneficial/pathogenic bacterium

**Figure 2:**
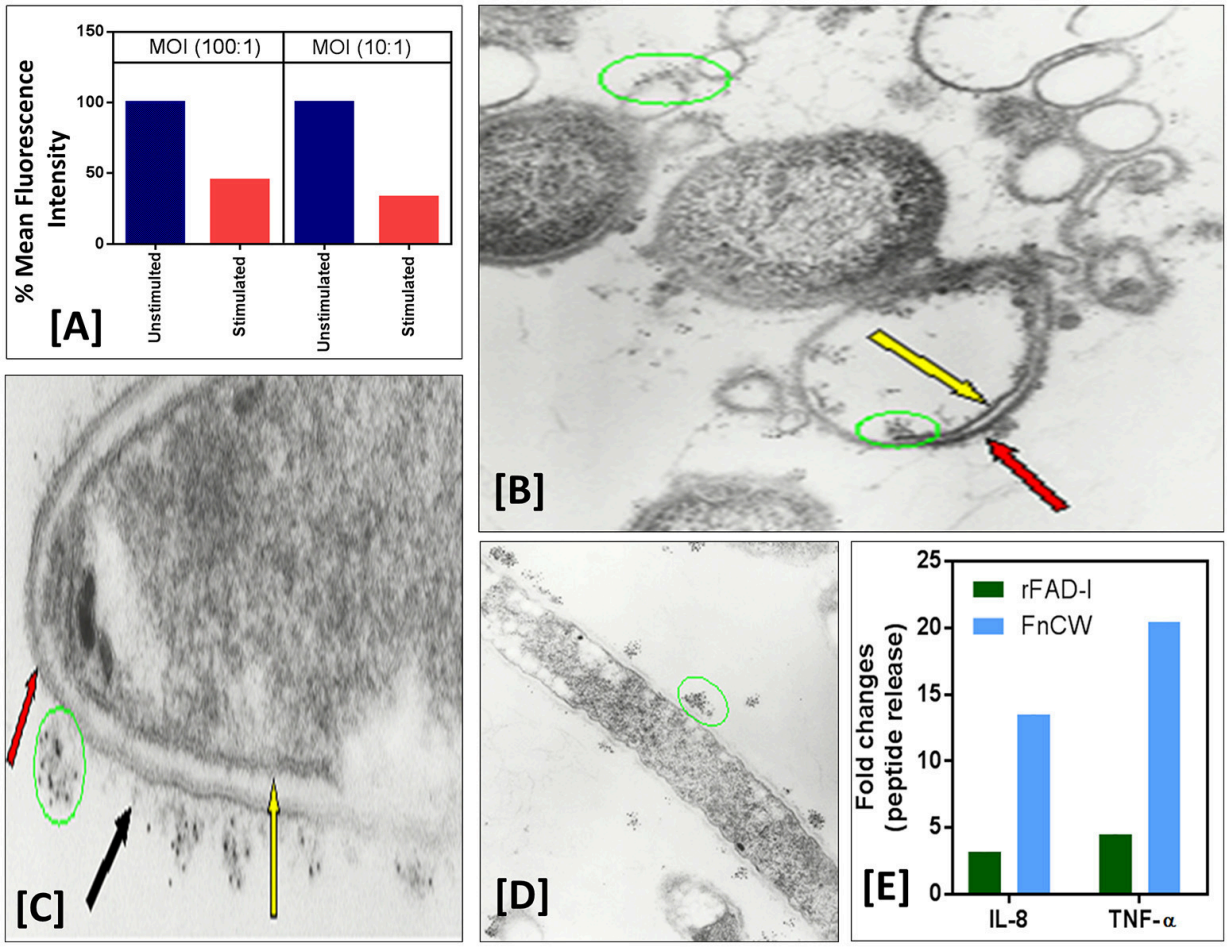
**(A)** *F. nucleatum* stimulation of normal human oral epithelial cells (HOECs) confers protection against *P. gingivalis* invasion. Semi-confluent (80%) monolayers of HOECs were either unchallenged or challenged with *F. nucleatum* cell wall fraction (FnCW) (10 μg/ml) for approximately 18 hrs. *P. gingivalis* was then added at a multiplicity of infection (MOI) of 10:1 or 100:1, 90 min, 37°C, 5% CO_2_. After 1 h incubation with gentamycin and metronidazole, cells were harvested and subjected to flow cytometric analysis. Results revealed a 54.3 and 67.2% reduction in *P. gingivalis* invasion for the 100:1 and 10:1 MOI's respectively, when compared to non *F. nucleatum* challenged HOECs. **(B–D)** Immunogold transmission electron microscopy (TEM) of *F. nucleatum* and *P. gingivalis* incubated with rhBD-2. Overnight cultures of *F. nucleatum* (ATCC strain 25586) and *P. gingivalis* (ATCC strain 33277) (1.6 × 10^9^ cells/ml) were incubated with recombinant hBD-2 (rhBD-2) (10 μg/ml), 3 h, 37°C anaerobically, and embedded in 1.5% low gel temperature agarose (Bio-Rad), respectively. Samples were fixed, 10 min at room temperature with 1% formaldehyde and 0.1% glutaraldehyde in 1x HEPES-buffered saline (pH 7.4), followed by washing 3X with 1X phosphate buffered saline (PBS) containing 0.05M glycine to block glutaraldehyde groups remaining on the cell surface. Samples were blocked in PBS with 1% BSA (bovine serum albumin; PBS-BSA), followed by incubation with goat anti-hBD-2 antibody (Cell Sciences, Canton, MA) (1:100) in PBS-BSA, 2 h, room temperature. After washing, samples were incubated, 2 h, in 5 nm gold-conjugated rabbit anti-goat IgG (BB International) (1:30) in PBS-BSA. To stabilize the gold particles, the samples were fixed with glutaraldehyde and post-fixed in 1% osmium tetroxide for 1 h. Samples were then block-stained in 0.5% of aqueous uranyl acetate, dehydrated in ascending concentrations of ethanol and embedded in Epon 812. Ultrathin sections were then stained with 2% uranyl acetate in 50% methanol and lead citrate, and examined in an electron microscope (Model Zeiss CEM902, Oberkochen, Germany). Black arrow points to *F. nucleatum* amorphous-like structures emanating from the organism's outer membrane to which immunogold labeled rhBD-2 (green circles) is sequestered, keeping it from interacting with the bacterium's outer membrane (red arrows) **(C)**. Yellow arrow points to intact *F. nucleatum* cytoplasmic membrane. Extensive *P. gingivalis* cellular debris of outer (red arrow) and cytoplasmic membrane (yellow arrow) with rhBD-2 sequestered to these structures (green circles) are also shown **(D)**. **(E)** Semi-confluent HOECs were treated with 10 μg/ml of either FnCW or recombinant FAD-I (rFAD-I) for 18 h. Levels of IL-8 and TNF-α in the supernatants were measured by ELISA (R&D systems, MN, US). Fold change in IL-8 and TNF- α released by each of the treatment compared to untreated cells were calculated.

We and others have shown that not only can whole *F. nucleatum* (ATCC-25586) organisms induce hBD-2, but that purified *F. nucleatum* cell wall is sufficient to promote defensin induction (Krisanaprakornkit et al., 2002; Gupta et al., 2010). To test if HOECs expressing hBDs are protected from bacterial invasion, we compared HOECs, after challenge with *F. nucleatum* cell wall, with unchallenged HOECs for levels of *P. gingivalis* invasion. HOECs that were *F. nucleatum* cell wall pretreated HOECs that were then challenged with fluorescently labeled (Syto62) *P. gingivalis* revealed a greater than 65% resistance to *P. gingivalis* invasion compared to cells not pretreated with *F. nucleatum* cell wall (Figure 2A). We surmise that the inhibition in invasion is due to *P. gingivalis* sensitivity to hBD-2 released from *F. nucleatum* challenged HOECs, as EM photographs demonstrate killing of *P. gingivalis* by rhBD-2 (Figures 2B–D).

Although we propose that hBD-2 is playing a major role in mucosal protection, we cannot rule out the possibility of synergy with other inducible epithelial cell derived AMPs. We (Ghosh et al., 2011) and others (Yin and Dale, 2007) have shown that *F. nucleatum* induces CCL20 (Mip3α), another AMP of epithelial origin. Moreover, hBD-2 itself has the ability to induce CCL20 mRNA (Yin and Dale, 2007) and peptide release from HOECs (Ghosh et al., 2011). Recently, we discovered that *F. nucleatum* can also promote the expression of the cathelicidin AMP, LL-37, in the presence of Vitamin D3 (data not shown). Interestingly, *F. nucleatum* biofilm has been shown to induce multiple AMPs in a dento-epithelial organotypic culture model (Gursoy et al., 2012). These data, collectively, suggest that *F. nucleatum* induces multiple AMPs that could be working in unison to protect the host from non-beneficial/pathogenic bacteria.

## Identifying the fusobacterial AMP-inducing agent

Biochemical fractionation of the cell wall from *F. nucleatum* (ATCC 25586), followed by a molecular biological approach (Gupta et al., 2010) revealed that the product of gene *FN1527* (*F. nucleatum* strain ATCC 25586; Kapatral et al., 2002) is responsible for the induction of hBD-2 in HOECs through interaction with toll-like receptors (TLRs) 1/2 and 2/6 (Bhattacharyya et al., 2016). We now refer to that product as FAD-I (for *F**usobacterium* Associated Defensin Inducer) (Gupta et al., 2010). A notable attractive property that we discovered for FAD-I, is its' ability to induce additional AMPs by HOECs; e.g., CCL20 (MIP-3α), a chemokine that has microbicidal activity (Ghosh et al., 2011), and the cathelicidin LL37 (unpublished observation), that requires the presence of Vitamin D for activation (McMahon et al., 2011). Like hBD-2 and CCL20, LL37 has both antimicrobial and immunoregulatory properties (Bowdish et al., 2006; Dürr et al., 2006).

Uehara et al. (2007) demonstrated that TLRs and (NOD-like receptors; NLRs), are the functional receptors on and in human cells responsible for inducing anti-bacterial responses without concomitant inflammatory responses. We currently established a new line of investigation with FAD-I, to show that it promotes expression of multiple innate immune defense molecules in mucosal epithelium while not provoking a pro-inflammatory cytokine response. Post FAD-I- and FnCW-treated transcriptome analyses of HOECs revealed differential induction of several cytokines/chemokines by FAD-I, when compared to FnCW, which was further confirmed by ELISA, demonstrating that FAD-I induced substantially lower levels of IL-8 and TNF-α release by HOECs, when compared to FnCW treated HOECs (Figure 2E).

## What can all of this mean?

Commensal bacteria are valuable to the host by inhibiting pathogens from colonizing a microbial niche and/or by secreting antimicrobial compounds. They also offer protection by continuously stimulating mucosal surfaces to express and release AMPs, capable of eradicating opportunistic/pathogenic organisms (Boman, 2000). Herein, we present a novel strategy by the commensal organism *F. nucleatum*, whereby this ubiquitous Gram-negative colonizer of the human oral cavity induces hBD-2 expression in oral epithelial cells; along with expression and release of CCL20 and LL37, which, as a consequence, confers protection to the cells from the opportunistic pathogen *P. gingivalis. P. gingivalis*, which fails to induce hBD-2, is sensitive to this AMP, while *F. nucleatum* strains that induce it are resistant. This strategy may be an expression of adaptive mutualistic co-evolution between *F. nucleatum* and the human host. The data presented are consistent with the notion that an organism that induces the production of a host antimicrobial agent may be resistant to it, while a sensitive one may have evolved mechanisms to avoid induction of this agent. It should be noted that not all *F. nucleatum* subspecies induce hBD-2 equally well (Bhattacharyya et al., 2016). Similar observations have been reported for other organisms; e.g., the incubation of epithelial cells with *Aggregatibacter actinomycetemcomitans strain* ATCC-29523 does not increase the hBD-3 expression in host cells, while the strain ATCC strain-33384 does (Vankeerberghen et al., 2005).

We hypothesize that *F. nucleatum*'s strategy of using the host to kill other bacteria is a mechanism that may contribute to homeostasis in body sites that are constantly challenged with microbes. Interestingly, while AMPs from non-human sources, such as protegrins from pigs, cecropins from insects and pigs, are effective in killing *F. nucleatum* at low micromolar concentrations, AMPs from human sources such as α-defensins, are ineffective against *F. nucleatum*, even at much higher concentrations (Miyasaki et al., 1998). In a series of elegant experiments, *F. nucleatum* was shown to respond to increased α-defensin concentrations by increasing its membrane thickness and decreasing membrane permeability, suggesting a mechanism of defense against these AMPs (Keskin et al., 2014; Musrati et al., 2016). Since our present findings demonstrate that *F. nucleatum* is also resistant to β-defensins, the collective information may be suggesting that evolution has sanctioned a close association between *F. nucleatum* and the human host.

Previous studies by others and our data to date support the principle that *F. nucleatum* has evolved to create a “heightened state of readiness” of the epithelium it inhabits without promoting notable inflammatory cytokine responses. This does not necessarily mean that *F. nucleatum* maintains the similar degree of “symbiosis” in other body sites, as the organism has been reported to infect vulnerable sites outside the oral cavity (Bolstad et al., 1996; Williams et al., 2003), and more recently has been shown to be associated with esophageal (Yamamura et al., 2016), colon (Castellarin et al., 2012), and oral cancers (Schmidt et al., 2014; Al-Hebshi et al., 2017). We therefore hypothesize that *F. nucleatum* may utilize its' resistance to defensins as a “virulence strategy” in its ability to promote extra-oral infections by invading epithelial cells (Han et al., 2000), and possibly in association with systemic complications (Bultink et al., 1999; Bauer et al., 2000; Han et al., 2004).

The “Dr. Jekyll/Mr. Hyde” scenario depicted for *F. nucleatum*, opens the perspective for additional research to more fully understand what promotes *F. nucleatum's* beneficial behavior vs. contributing to/exploiting disease. The role the beneficial factor, FAD-I, plays in each of these scenarios would be exceedingly interesting to discern.

The ability of FAD-I to promote AMP induction without excessive pro-inflammation is unique amongst bacterial lipoproteins, since studies of lipoproteins isolated from other gram-negative bacteria, primarily pathogenic ones, have shown activation of host related inflammation (Kovacs-Simon et al., 2011; Wilson and Bernstein, 2016; Dennehy et al., 2017; Wang et al., 2017). Therefore FAD-I is attractive when proposing an agent to protect vulnerable mucosal sites from dysbiosis and chronic inflammation. Recently, histone deacetylase (HDAC) inhibition was shown to enhance AMP expression without concomitant inflammatory cytokine expression (Fischer et al., 2016). The authors hypothesized that two sets of genes; i.e., AMPs vs. inflammatory cytokines, may obey differential regulatory rules when the host is subjected to a pathogenic microbe vs. tolerating commensal microbiota. Could *F. nucleatum* be a key commensal that promotes “physiological” inflammation, tweaking the host mucosa to generate physiological levels of innate response agents that contribute to homeostasis? Could FAD-I have the ability to promote epigenetic changes directly or induce an HDAC inhibitor that then, in conjunction with FAD-I contribute to selective “physiologically” relevant innate responses?

## Conclusions and future perspectives

Conventional antibiotics are becoming less effective owing to the growing problem of resistance. There is growing interest developing antimicrobial agents, on the basis of natural ones, such as hBDs, as human therapeutics to eradicate antibiotic resistant bacteria. Unlike prokaryotic originating antibiotics that bacteria can resist over time, AMPs is surprisingly rare and difficult to generate. The main problems impeding the development of AMPs as systemic therapy include the observations that many of them, although active *in vitro*, are only effective in animal models of infection when administered at toxic doses; reflecting an unacceptable margin of safety. Synthetic AMPs, engineered to be more cationic and more microbicidal are also more cytotoxic. Finally, the cost associated with generating synthetic AMPs is prohibitive and the pharmacokinetic question of delivering the exogenous AMP to a body site is quite challenging. From our experience, FAD-I, is recombinantly produced in *E. coli* (Gupta et al., 2010; Bhattacharyya et al., 2016), followed by LPS decontamination, at a present cost of $600/mg. Molecules like FAD-I, that can promote endogenous AMP production, have the potential of reducing dysbiosis-driven diseases at a time when resistance to antibiotics is rising. We therefore propose that a paradigm shifting therapeutic strategy could be developed by exploiting AMP inducing bacterial molecules that promote endogenous AMP production in vulnerable body sites, without promoting localized inflammation.

## Ethics statement

Tissue acquisition for primary cell isolation was conducted in accordance with our Institutional Review Board (IRB)-approved protocol (NHR-15-19) for the use of discarded tissue.

## Author contributions

SG and AW: Wrote the manuscript; ZF and HF: Performed the experiments; RL and TM: Edited the manuscript.

### Conflict of interest statement

The authors declare that the research was conducted in the absence of any commercial or financial relationships that could be construed as a potential conflict of interest.
